# Characterization of ATP7A missense mutants suggests a correlation between intracellular trafficking and severity of Menkes disease

**DOI:** 10.1038/s41598-017-00618-6

**Published:** 2017-04-07

**Authors:** Tina Skjørringe, Per Amstrup Pedersen, Sidsel Salling Thorborg, Poul Nissen, Pontus Gourdon, Lisbeth Birk Møller

**Affiliations:** 1 Applied Human Genetics, Kennedy Center, Department of Clinical Genetics, Copenhagen University, Rigshospitalet, Glostrup Denmark; 2 Section of Neurobiology, Biomedicine group, Institute of Medicine and Health Technology, Aalborg University, Aalborg, Denmark; 3 Department of Biology, August Krogh Building, University of Copenhagen, Universitetsparken 13, DK-2100 Copenhagen OE, Denmark; 4 Centre for Membrane Pumps in Cells and Disease-PUMKIN, Aarhus University, Department of Molecular Biology, Gustav Wieds Vej 10C, DK-8000 Aarhus C, Denmark; 5 Department of Biomedical Sciences, University of Copenhagen, Blegdamsvej 3B, DK-2200 Copenhagen, Denmark; 6 Department of Experimental Medical Science, Lund University, Sölvegatan 19, SE-221 84 Lund, Sweden; 7 Department of Science, Systems and Models, Roskilde University, Universitetsvej 1, DK-4000 Roskilde, Denmark

## Abstract

Menkes disease (MD) is caused by mutations in *ATP7A*, encoding a copper-transporting P-type ATPase which exhibits copper-dependent trafficking. ATP7A is found in the Trans-Golgi Network (TGN) at low copper concentrations, and in the post-Golgi compartments and the plasma membrane at higher concentrations. Here we have analyzed the effect of 36 *ATP7A* missense mutations identified in phenotypically different MD patients. Nine mutations identified in patients with severe MD, virtually eliminated ATP7A synthesis, in most cases due to aberrant RNA splicing. A group of 21 predominantly severe mutations led to trapping of the protein in TGN and displayed essentially no activity in a *yeast-*based functional assay. These were predicted to inhibit the catalytic phosphorylation of the protein. Four mutants showed diffuse post-TGN localization, while two displayed copper dependent trafficking. These six variants were identified in patients with mild MD and typically displayed activity in the yeast assay. The four post-TGN located mutants were presumably affected in the catalytic dephosphorylation of the protein. Together these results indicate that the severity of MD correlate with cellular localization of ATP7A and support previous studies indicating that phosphorylation is crucial for the exit of ATP7A from TGN, while dephosphorylation is crucial for recycling back to TGN.

## Introduction

Menkes disease (MD; OMIM: 309400), including the milder form Occipital Horn Syndrome (OHS; OMIM: 304150), is a rare (1:300.000), X-linked, multisystemic lethal disorder of copper metabolism linked to mutations in the *ATP7A* gene (OMIM: 300011). The symptoms of MD derive from a lack of dietary copper absorption and copper transfer across the basolateral membrane of intestinal enterocytes into the portal circulation, and from impaired reabsorption of copper in the kidney. This leads to accumulation of copper in intestinal cells and kidney, and lack of copper in vital organs such as heart, liver and brain, and reduced activity of essential cuproenzymes^1–3^. MD can be divided into three subclasses based on phenotype and progression. The severe classical form (observed in 90–95% of MD patients) is characterized by progressive neurodegeneration, connective tissue abnormalities, distinctive “kinky” hair and death typically before the age of three years. Atypical mild MD is characterized by longer survival and/or milder symptoms of the affected patients. OHS is mainly characterized by connective tissue manifestations and is the mildest form^1^. Recently also two unique mutations have been shown to cause isolated adult-onset spinal muscular atrophy (OMIM: 300489)^4^.

**Figure 1:**
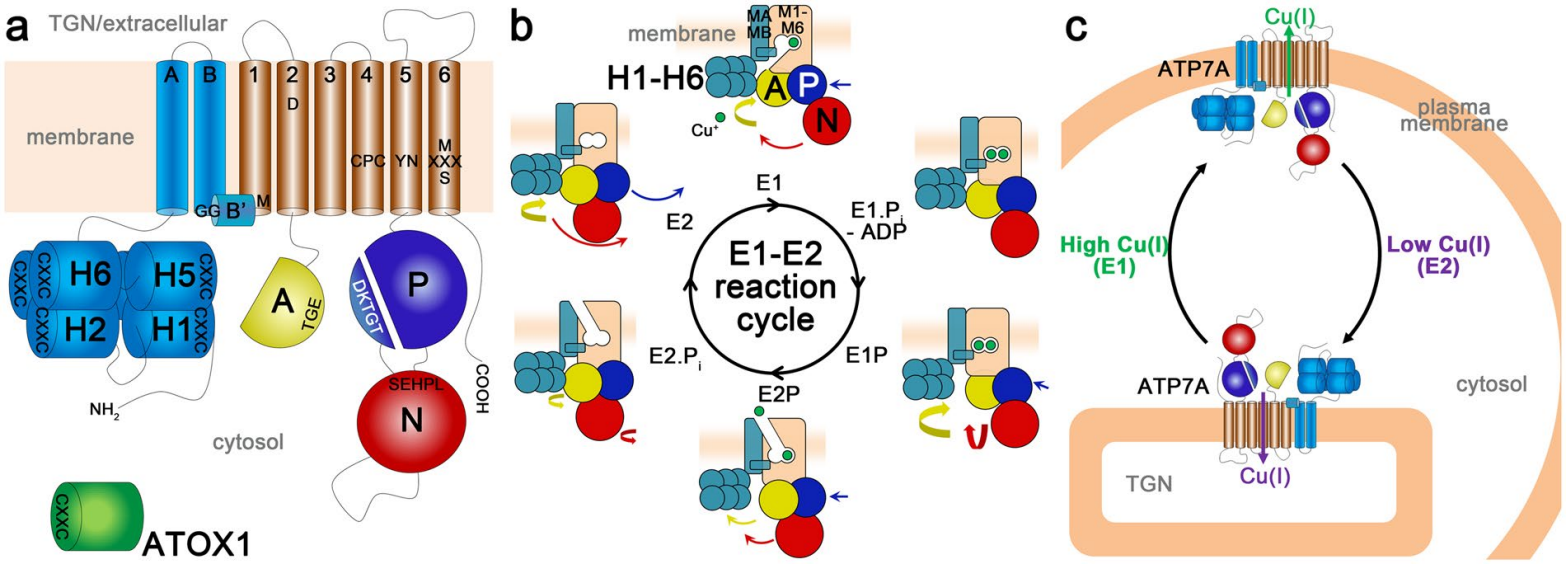
Topology and reaction cycle of ATP7A. (**a**) Topology of ATP7A. ATP7A, and other P-type ATPases consist of three cytoplasmic domains, nucleotide binding (N, red), phosphorylation (P, blue) and dephosphorylation/actuation (A, yellow). The transmembrane domain encompasses eight membrane-spanning segments (TM), two class I specific (TMA-TMB, cyan) and six conserved segments (TM1-6, wheat). The N-terminus contains six class-specific metal-binding domains (H1-H6, cyan). The copper-donating chaperone ATOX1 (green) and conserved motifs are shown. The non-cytosolic part of ATP7A is located in the TGN or in the extracellular milieu, due to copper-dependent trafficking. (**b**) The Albers-Post (E1-E2) reaction cycle of ATP7A and other Cu-transporting P-type ATPases. The domains are colored as described above, and copper ions are shown in green. Phosphorylation events in the intracellular domains drive large conformational changes that permit alternating access to transport sites in the membrane about 50 Å from the ATP-targeted catalytic aspartate. A high-affinity state (E1) binds copper and enters an occluded state, which then undergoes phosphorylation (E1.Pi-ADP). Completion of this event (E1P) triggers release of the ion, establishing an outward-facing, low-affinity state (E2P). Release of inorganic phosphate (E2.Pi) yields the fully dephosphorylated conformation (E2), which is followed by restoration of the inward-facing conformation (E1) that initiates a new reaction cycle. (**c**) Proposed cellular trafficking of ATP7A as an effect of copper concentration. At low cellular copper concentrations the wild-type ATP7A is located in the Trans-Golgi Network (TGN), whereas at higher intracellular copper levels, the steady state distribution of ATP7A shifts to the plasma membrane (and cytosolic vesicles, not shown).

ATP7A belongs to the P-type ATPase family of ATP-driven membrane pumps that maintain electrochemical gradients as well as cationic and lipid homeostasis. P-type ATPases share a structural core containing a transmembrane (TM) domain responsible for transport, and three soluble domains - N, P and A - required for nucleotide binding, phosphorylation, and dephosphorylation, respectively (Fig. 1a). Copper transporting members of the family, including ATP7A and bacterial CopA proteins, have eight membrane spanning helical segments (TMA, TMB, TM1-TM6), and typically one or more metal-binding domains (MBD) at the amino-terminus with metal-binding CXXC motifs. The mammalian P-type ATPases, ATP7A and ATP7B, the latter mutated in Wilson’s disease, have six MBDs in sequential order^5^.

The catalytic cycle of the ATP7A protein is associated with four principal corner stone reaction intermediates (E1, E1P, E2P and E2, Fig. 1b) with considerably different three-dimensional shapes due to structural rearrangements. ATP7A function requires cytosolic copper delivered to the TM domain^6–8^. Copper is donated from copper chaperones such as ATOX1^9, 10^. The E1 state of the protein binds copper with high affinity within the TM domain^11, 12^. Copper-binding and ATP-recognition allow for auto-phosphorylation of the invariant D1044 in the P-domain leading to the E1P state and occlusion of copper in the TM domain. Completion of phosphorylation triggers large conformational changes that affect access to the ion-binding residues permitting copper release to the non-cytosolic side, reaching the E2P state^13–16^. Auto-dephosphorylation of the protein to the E2 conformation follows, which then shifts to the E1 state to initiate a new reaction cycle^17^. The ^875^TGE motif of the A-domain is important for auto-dephosphorylation^15^.

In mammals, trafficking of ATP7A is essential for proper copper homeostasis. At low cellular copper concentrations wild-type ATP7A is located in the Trans-Golgi Network (TGN), whereas at higher intracellular copper levels, the steady state distribution of ATP7A shifts to cytosolic vesicles and to the plasma membrane (Fig. 1c)^18, 19^. In the TGN, the ATP7A protein is essential for the delivery of copper to cuproenzyme biogenesis, and in the plasma membrane ATP7A is responsible for extrusion of excess copper from the cell^19, 20^. The copper-mediated trafficking response is reversible; if the intracellular copper level is reduced ATP7A returns to TGN^19^.

Although the mechanism behind the copper-dependent trafficking of ATP7A is far from fully understood, it has been demonstrated that several elements of the ATP7A protein play important roles. A 38 amino acid sequence containing transmembrane segment 3 was found to be essential for localization to the Golgi complex^21^. At least one MBD needs to be present for correct copper-dependent trafficking^22^. The dileucine motifs, 1487LL and 1459LL within the cytosolic carboxy terminal, have been shown to be essential for TGN localization by playing a role in the retrieval of ATP7A from the plasma membrane^23^. It has recently been demonstrated that Adaptor Protein complexes 1 and 2 interact physically with ATP7A, probably through the 1478LL motif, and this interaction might mediate the trafficking^24^. Copper-responsive kinase phosphorylation of S1469 and constitutive phosphorylation of S1432 have also been demonstrated to regulate the trafficking of ATP7A^25^. Importantly, phosphorylation of the catalytic D1004 has been shown to play an important role in copper-induced re-localization of ATP7A from TGN and the subsequent dephosphorylation important for retrieval^13^.

Few ATP7A variants in man and mice have previously been investigated for the effect on copper-dependent trafficking^18, 26–28^. By studying a large number of missense mutations, all of which hamper copper homeostasis observed in patients with different phenotypes, it is possible to achieve a greater clarification of the complex copper-dependent trafficking process. As a consequence of the E1-E2 reaction cycle and copper-dependent localization of ATP7A, there are a number of ways by which missense mutations may hamper copper homeostasis. Different steps in the reaction cycle such as copper-binding or -release, phosphorylation or dephosphorylation may be prevented. In addition, copper-dependent trafficking may be impaired. At the same time, missense mutations may also influence biogenesis via mRNA splicing, mRNA stability, protein folding or protein stability.

To shed further light on missense mutations in general and the role of MD mutations in particular, we here investigated the effect of 36 missense mutations the *ATP7A* gene with regard to i) the splicing of *ATP7A* pre-mRNA, ii) levels of *ATP7A* transcript and ATP7A protein product, iii) the intracellular localization of the mutated ATP7A protein and iv) *in vivo* copper transport capacity.

We find that a significant fraction of the mutations lead to the absence or severely reduced amounts of ATP7A protein, rather than affecting the function of the ATP7A protein *per se*. With respect to the mutations which allow the production of ATP7A protein, most of the mutations affect residues that are implicated in the ATPase-coupled transport function, and abolish copper-dependent trafficking of ATP7A. Enzymatic activity and trafficking thus appear to be coupled processes for ATP7A.

Our work reveals that severe mutations, predicted to inhibit the catalytic phosphorylation of the protein led to trapping of the protein in TGN, whereas milder mutations, predicted to inhibit the catalytic dephosphorylation of the protein led to diffuse post-TGN localization of the protein. Thus, the results suggest a correlation between the severity of the mutations and the cellular localization of the protein.

## Results

A library of cultured fibroblasts from 36 different Menkes/OHS patients (24 with the classical MD phenotype, ten with a typical MD, one with OHS and one with a mixed manifestation), each carrying a missense mutation in the *ATP7A* gene^29^, was used for investigating the molecular cause of MD in these patients.

### Biochemical diagnosis confirmed Menkes disease

**Figure 2:**
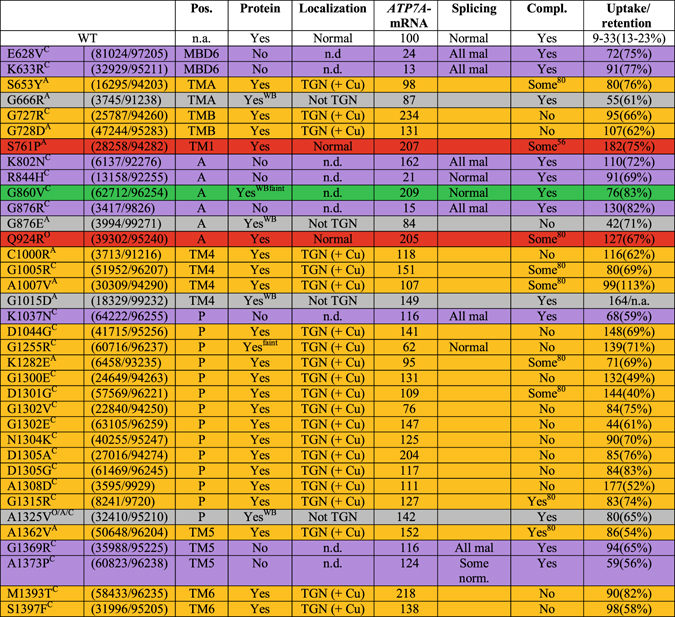
Summary. The color represents the mutational effect on ATP7A localization: Normal trafficking of the protein (blue); retention in post-Golgi compartments (grey); retention in the trans-Golgi network (orange); not certain (green); no detectable protein (purple). ^“A”^ denote atypical, ^“O”^ OHS, and ^“C”^ classical phenotype. Pos: indicates affected region (MBD, TM, A, P). Protein: Indicates whether protein was detectable with IF “Yes” or WB only “Yes^WB^”. Localization: Location of ATP7A always in TGN even in the presence of Cu is designated by “TGN(+Cu)”. Never located in TGN is designated by “not TGN”; Location in the TGN only the absence of copper is designated by “normal”. No detectable protein “n.d”. ATP7A-mRNA: indicates % transcript level compared to transcript level in control fibroblasts. Splicing: indicates normal splicing “Normal” or aberrant splicing “All mal”. Empty boxes, indicate “not investigated”. Compl: indicates ability to complement the ccc2Δ yeast strain. “Some^56^” and “Some^80^”, indicate some complementation after 56 hours and 80 hours, respectively. “Yes^80^” indicates full complementation after 80 hours. If no hours are indicated, it means >56 hours. Uptake/retention: Uptake indicates the amount of radioactive copper accumulated after 20 hours incubation (^64^Cu/mg protein/20 hours). Retention indicated the amount in % of total accumulated ^64^Cu retained after subsequent 24 hours in the absence of radioactive copper.

The copper-uptake capacity (^64^Cu uptake/mg protein/20 h) of fibroblasts from the 36 patients was analyzed and found to be higher, whereas the excreting capacity (% of total ^64^Cu-uptake retained after 24 h) was lower when compared to control fibroblasts, indicating Menkes disease in all cases (Fig. 2).

### Copper-regulated trafficking of ATP7A variants in MD fibroblasts is compromised

**Figure 3:**
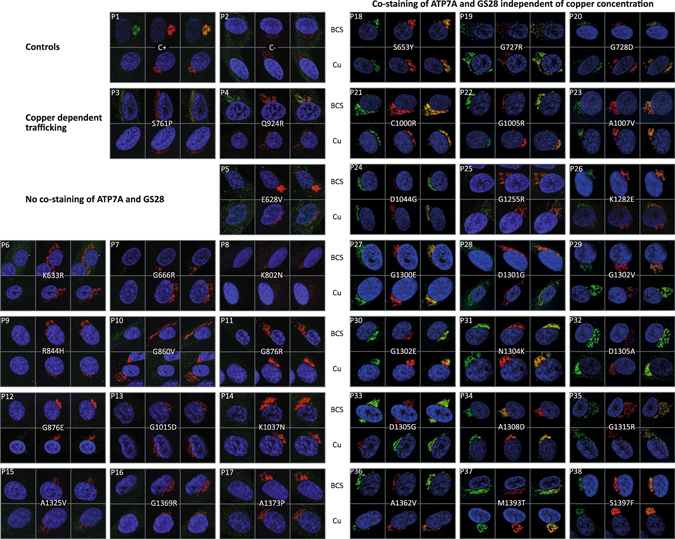
Cellular investigation of endogenous ATP7A protein in MD fibroblasts by indirect immunofluorescence (IF). MD fibroblasts with various missense mutations were stained with primary antibodies against ATP7A (green; position 1) and the Golgi specific marker GS28 (red; position 2), respectively. Also a merge picture is shown (position 3). The nuclei were counterstained with DAPI. Copper-dependent trafficking was investigated in the presence of BCS (upper panel) and CuCl_2_ (lower panel), respectively. The pictures are divided into groups defined by the effect of the mutation on cellular location of the ATP7A protein. P1: Normal control cells (C+). P2: ATP7A negative control cells (C−). P3–P4: Copper dependent trafficking. P5-P17: No ATP7A signal was detected. P18–P38: Hampered copper-induced trafficking from TGN.

Fibroblasts from the 36 patients were evaluated for the presence and localization of ATP7A protein by immunofluorescence (IF) (Fig. 3). Fibroblasts from a healthy person were included as a positive control, whereas fibroblasts from a MD patient with a deletion of exons 3–23 (c.121−?_8333+?del) in ATP7A were used as a negative control. Fibroblasts were either treated with CuCl_2_ or the copper chelator Bathocuproine disulphonate (BCS) to create a milieu of excess versus low amounts of bio-available copper.

In positive control fibroblasts, ATP7A showed a copper-dependent localization. In the presence of BCS, ATP7A is located in TGN, and is partly co-localized with the TGN GS28 SNARE protein, whereas in the presence of copper, distinct localization was replaced by a diffuse, cytoplasmic, and very weak ATP7A signal (Fig. 3, Panel P1; Supplementary Fig. 1) in accordance with previous studies^17^. The loss of a TGN-signal for ATP7A - when conditions change from low to high copper levels - indicates copper-induced trafficking. No ATP7A signal was detected in the negative control cells (C−) under any conditions (Fig. 3, Panel P2; Supplementary Fig. 2).

A copper dependent trafficking pattern was only observed for ATP7A with the predicted amino acid substitutions S761P^A^ or Q924R° (Fig. 3, Panels P3 and P4, ^A^ and ° denote atypical and OHS phenotypes, respectively; Supplementary Figs 3 and 4). Although copper dependent trafficking was observed, the pattern of S761P^A^ and Q924R° was more diffuse in the presence of BCS, compared to the wild type ATP7A (C+), with staining in the cytoplasm in addition to TGN. The degree of co-localization with GS28 of Q924R° was however on par with the wild type ATP7A, whereas the co-localization of S761P^A^ with GS28 was reduced compared to wild type ATP7A, indicating mis-localization to other areas of TGN or other nearby located compartments of the endomembrane systems (Supplementary Figs 3 and 4). The Pearson's correlation R values, (co-localization analysis tool, Coloc 2; Fiji) in the presence of BCS/Cu respectively were 0.41/0.06 for Q924R°, 0.18/−0.06 for S761P^A^, and 0.51/0.07 for wild type ATP7A. The R values for negative control cells (C−) in the presence of BCS/Cu respectively were −0.02/−0.19 (Supplementary Table 1). An R value of 1 corresponds to total positive correlation, 0 corresponds to no correlation, and −1 corresponds to total negative correlation.

In contrast, when looking at ATP7A with the 21 predicted substitutions: S653Y^A^, G727R^C^, G728D^A^, C1000R^A^, G1005R^C^, A1007V^A^, D1044G^C^, G1255R^C^, K1282E^A^, G1300E^C^, D1301G^C^, G1302V^C^, G1302E^C^, N1304K^C^, D1305A^C^, D1305G^C^, A1308D^C^, G1315R^C^, A1362V^A^, M1393T^C^, and S1397F^C^ (Fig. 3, Panels P18–P38, ^C^denote classical phenotype), copper-dependent trafficking was hampered because ATP7A was localized in the TGN regardless of the copper concentration. The Pearson’s correlation R values for co-localization with GS28 were between 0.30 and 0.73 for all cultures with no copper dependent differences, in agreement with no copper dependent trafficking. The signal for G1255R^C^ was however very faint (Fig. 3, Panel P25; Supplementary Table 1).

The remaining 13 mutants (E628V^C^, K633R^C^, G666R^A^, K802N^C^, R844H^C^, G860V^C^, G876R^C^, G876E^A^, G1015D^A^, K1037N^C^, A1325V^A/^ ^O^ ^/C^, G1369R^C^ and A1373P^C^) did not reveal any convincing ATP7A signal regardless of the treatment (Fig. 3, Panels P5 to P17). The results are summarized in Fig. 2.

### Western blotting confirmed that ATP7A protein is present in a fraction of the IF negative cultures

The ATP7A protein is difficult to visualize by IF staining unless it accumulates in a physically distinct cellular compartment such as TGN. The inability to detect ATP7A protein by IF may therefore not only be due to amino acid substitutions which compromise ATP7A accumulation, but could also be due to substitutions which abolish distinct TGN-location.

**Figure 4:**
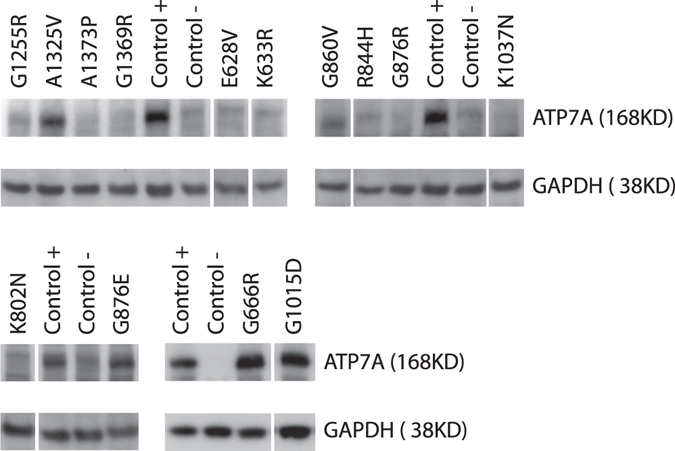
Western blots of lysates from MD fibroblasts. Western blots of lysates from MD fibroblasts with no detectable ATP7A protein when analysed by Immunofluorescence (IF). The specific *ATP7A* missense mutations are indicated above the lanes. Control + lysate from normal fibroblasts. Control − lysate from MD fibroblasts containing a big deletion of the *ATP7A* gene (exon 3–23 deleted). The protein product of the housekeeping gene *GAPDH* was used as an internal control for correct loading of 25 μg protein in each lane. The staining of ATP7A and GAPDH was performed on one membrane cut in two. Gaps indicate separate gels. Small gaps indicate that the gel was cut in producing the figure.

The fibroblast cultures with no IF-detectable ATP7A were therefore examined by Western blot analysis (WB) to assess whether any ATP7A protein was present (Fig. 4). We included the G1255R^C^ substitution, with very faint IF signals, to compare the WB and IF results.

The band intensity for ATP7A in cells with the substitutions G666R^A^, G876E^A^, G1015D^A^ and A1325V^A/O/C^ corresponded to the band intensity of the wild-type positive control, indicating that these four amino acid substitutions did not affect protein accumulation, but hamper their localization to TGN.

The band intensity of accumulated G860V^C^ and G1255R^C^ protein was slightly reduced, making it difficult to determine the cellular localization of G860V by IF. In contrast, no ATP7A protein was observed in cells predicted to express the eight variants E628V^C^, K633R^C^, K802N^C^, R844H^C^, G876R^C^, K1037N^C^, G1369R^C^ or A1373P^C^. The results are summarized in Fig. 2.

### Absence of ATP7A protein is in most cases due to aberrant splicing

Undetectable ATP7A protein may relate to severely reduced levels of correctly spliced mRNA, impaired protein synthesis or reduced protein stability. To distinguish between these possibilities, the level of *ATP7A* mRNA was determined in fibroblasts from all patients by real time PCR. Of the eight amino acid substitutions that prevented ATP7A accumulation, only four - E628V^C^, K633R^C^, R844H^C^ and G876R^C^ led to a reduced accumulation of *ATP7*A transcript (10–25% remained compared to the healthy control). Fibroblasts with the remaining four variants - K802N^C^, K1037N^C^, G1369R^C^ and A1373P^C^ - accumulated *ATP7A* mRNA on par with the healthy control. All twenty-eight additional *ATP7A* mutants had normal mRNA levels. The results are summarized in Fig. 2.

To test for erroneous *ATP7A* RNA splicing, reverse transcription-PCR (RT-PCR) was carried out on mRNA from fibroblasts encoding either i) E628V^C^, K633R^C^, R844H^C^ or G876R^C^ with a small amount of *ATP7A* transcript and no detectable ATP7A protein, ii) K802N^C^, K1037N^C^, G1369R^C^ or A1373P^C^, with a normal amount of *ATP7A* transcript, and no detectable ATP7A protein iii) G860V^C^ or G1255R^C^, with a normal amount of *ATP7A* transcript and a reduced amount of ATP7A protein. The PCR primers were designed to amplify the cDNA sequence covering the mutation-containing exon and several flanking exons.

**Figure 5:**
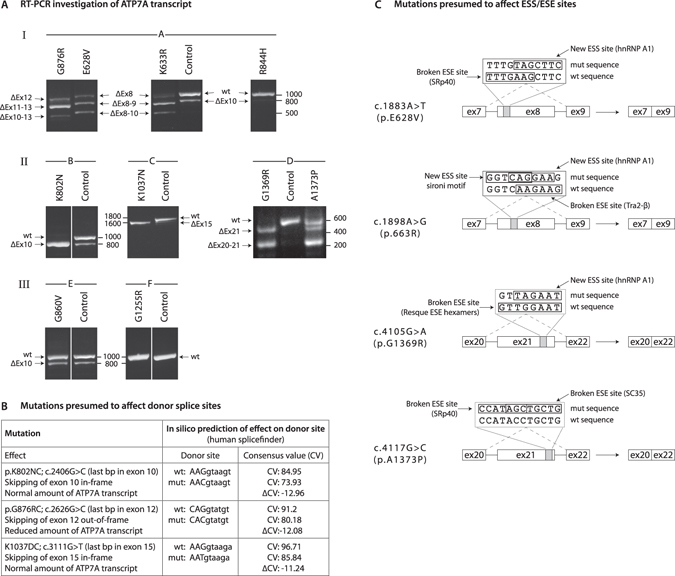
(**A**) RT-PCR investigation of *ATP7A* transcript. RNA from MD fibroblasts were subjected to RT-PCR spanning the exons with the missense mutations. Control samples are from a normal control. Size (in bp) of marker DNA fragments are indicated. The PCR products were separated by gel electrophoresis, purified and sequenced (Table 1). Primers are given in Supplementary Table 2. (I) No detectable ATP7A protein and a reduced amount of *ATP7A* transcript. A: PCR amplification of exons 7–14. E628V^C^ and K633R^C^ (mutations in exon 8); skipping of exon 8, exons 8–9 or exons 8–10. G876R^C^ (mutation in exon 12); skipping of exon 12, exons 11–13 or exons 10–13. R844H^C^ indistinguishable from the wild-type sample. (II) No detectable ATP7A protein and a normal amount of *ATP7A* transcript. B: PCR amplification of exons 7–14. K802N^C^ (mutation in exon 10); skipping of exon 10. C: PCR amplification of exons 12–23. K1037N^C^ (mutation in exon 15); skipping of exon 15. D: PCR amplification of exons 19–23. G1369R^C^ (mutation in exon 21); skipping of exon 21 or exons 20–21, and A1373P^C^ (mutation in exon 21); normal transcripts or skipping of exon 21 or exons 20–21. (III) Reduced amount of ATP7A protein and normal amount of *ATP7A* transcript. E: PCR amplification of exons 7–14. G860V^C^ indistinguishable from the wild-type sample F: PCR amplification of exons 17–23. G1255R^C^ indistinguishable from the wild-type sample. (**B**) Mutations presumed to affect donor splice sites. According to “human-Splicing Finder” (http://www.umd.be/HSF/) splice sites with consensus values (CV)’s higher than 80 are strong splice sites, whereas splice sites with CV’s between 65 and 70 are weak^30^. A relative change in CV (∆CV) of 10 percentage points relative to wild-type sites is predicted to attenuate splicing. (**C**) Mutations presumed to affect ESS/ESE sites. Disruption of ESE sequences and/or creation of an ESS sequences may lead to exon skipping. In agreement with the observed splicing pattern, the four base pair substitutions are predicted by the web-tools EX-SKIP (http://ex-skip.ing.cas.cz/)^32^ to lead to exon skipping as a result of increased ESS/ESE values. Selected motifs are illustrated.

Electrophoretic analysis of the RT-PCR products showed that the mutations predicting the substitutions G876R^C^, E628V^C^ and K633R^C^ (Fig. 5AI), K802N^C^, K1037N^C^, G1369R^C^ and A1373P^C^ (Fig. 5AII) all produced an RT-PCR pattern which differed from that of the wild-type. Except for A1373P^C^, sequence analysis of the PCR products did not reveal any normally spliced transcripts, but only mal-spliced transcripts in which different exons had been skipped. None of these exons, except for exon 10, were skipped in the healthy control. This concurs with previous observations that show that 10–15% of *ATP7A* transcripts in healthy cells skip exon 10^30^. In contrast, the RT-PCR band pattern obtained from R844H^C^, G860V^C^ or G1255R^C^ fibroblasts was indistinguishable from the wild-type sample (Fig. 5AI and AIII). Accordingly, sequence analysis confirmed that only normally spliced transcripts were present in these fibroblasts. These results are summarized in Table 1.

**Table 1:** Sequence of PCR products obtained by spanning RT-PCR.

MUTATIONS AFFECTING *ATP7A* mRNA AND/OR ATP7A PROTEIN LEVEL		
Mutation (Nucleotide numbers refer to *ATP7A* transcript ENSG00000269395 in Ensemble).	Transcripts in patient (effect on reading frame)	Transcripts in healthy control
**Mutations leading to decreased levels of** *ATP7A* **mRNA and no detectable ATP7A protein**		
E628V^C^ Exon 8; c.1883A > T	1) Skipping of exon 8 (out of frame, S624Mfs*35, PTT in exon 9 > NMD)	1) Normal splicing
	2) Skipping of exon 8–9 (in-frame)	2) Skipping of exon 10
	3) Skipping of exon 8–10 (in-frame)	
K633R^C^ Exon 8; c.1898A > G	1) Skipping of exon 8 (out-of-frame, S624Mfs*35, PTT in exon 9 > NMD)	
	2) Skipping of exon 8–9 (in-frame)	
	3) Skipping of exon 8–10 (in-frame)	
R844H^C^ Exon 10; c.2531 G > A	1) Wt (including the mutation)	
G876R^C^ Exon 12, c.2626 G > C last base in exon	1) Skipping of exon 12 (out–of-frame, S833Rfs*8, PTT in exon 13 > NMD)	
	2) Skipping of exon 11–13 (in-frame)	
	3) Skipping of exon 10–13 (in-frame)	
**Mutations leading to normal amounts of** *ATP7A* **mRNA, but no detectable ATP7A protein**		
K802N^C^ Exon 10, c.2406 G > C last base in exon	1) Skipping of exon 10 (in-frame)	1) Normal splicing
		2) Skipping of exon 10
K1037N^C^ Exon 15, c.3111 G > T last base in exon	1) Skipping of exon 15 (in-frame)	1) Normal splicing
G1369R^C^ Exon 21, c.4105 G > A	1) Skipping of exon 21 (out-of-frame, N1336Efs*26, PTT in exon 22 ≠ NMD)	1) Normal splicing
	2) skipping of exon 20–21 (out-of-frame)	
A1373P^C^ Exon 21, c.4117 G > C	1) Normal splicing (including the mutation)	1) Normal splicing
	2) Skipping of exon 21 (out-of-frame, N1336Efs*26, PTT in ex 22 ≠NMD)	
	3) Skipping of exon 20–21 (out–of-frame)	
**Mutations leading to normal amounts of** *ATP7A* **mRNA, but reduced amount of ATP7A protein**		
G860V^C^ Exon 12, c2579G > T	1) Normal splicing (including the mutation)	1) Normal splicing
	2) Skipping of exon 10 (in-frame)	2) Skipping of exon 10
G1255R^C^ Exon 19, c.3763 G > A	1) Normal splicing (including the mutation)	1) Normal splicing

^“A”^ denote atypical, ^“O”^ OHS, and ^“C”^ classical phenotype.

The nucleotide alterations leading to amino acid substitutions K802N^C^, G876R^C^ or K1037N^C^ define the 3′ end of exons 10, 12 and 15, respectively. Consequently, the observed aberrant RNA splicing may be due to poor 3′donor splice site recognition as predicted by the web-tool Human-Splicing Finder (http://www.umd.be/HSF/)^31^ (Fig. 5B).

In contrast, the erroneous splicing of the E628V^C^, K633R^C^, G1369R^C^ and A1373P^C^ mutated transcripts are most likely due to effects on exonic splicing regulatory elements, such as exonic splicing enhancers (ESE) or exonic splicing silencers (ESS)^32^. The effects are illustrated in Fig. 5C. Splicing defects caused by exonic missense mutations in *ATP7A* are part of the mechanism of pathogenesis in MD and have not been described previously. Only a single case, in which an exonic silent mutation was shown to lead to exon skipping, has recently been described^33^.

### Temperature affects accumulation of G860V

**Figure 6:**
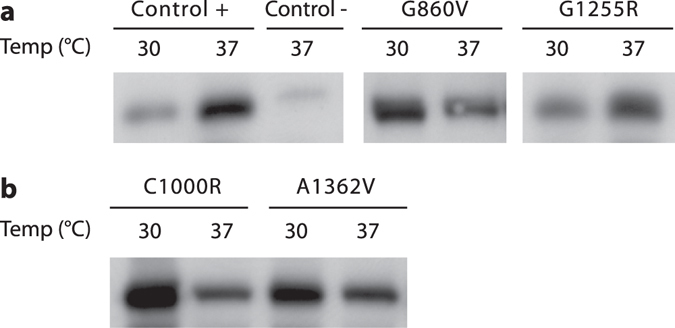
WB of lysates isolated from MD fibroblast cultures cultured at 30 °C and 37 °C, respectively. The MD fibroblasts contain different missense mutations as indicated. Control + is lysate from normal fibroblasts. Control − is lysate from MD fibroblast, containing a big deletion of the *ATP7A* gene (exon 3–23 deleted). The blots are representative for Western blots of three separate cultures that were harvested and prepared individually for each mutation and control. The Western blots were performed on separate occasions; therefore band intensity cannot be compared between different gels. (**a**) Mutations that lead to low levels of ATP7A in this study. (**b**) Mutations that are similar to or resemble the mutations analysed by Vonk *et al*.^34^. Gaps indicate that the gel was cut in producing the figure.

Neither reduced amounts, nor abnormal *ATP7A* mRNA splicing apparently account for the small amount of ATP7A protein accumulated in fibroblasts from patients with the G860V^C^ or G1255R^C^ substitution. Therefore, *in vivo* instability of these variants may explain the observed lack of protein accumulation. Increased protein accumulation at reduced growth temperatures has previously been described for unstable proteins with a compromised folding capacity. The accumulation of e.g. ATP7A^34^, ATP7B^35^ and P-type ATPase ATP8B1^36^ variants has previously been shown to increase in cells growing at 30 °C instead of 37 °C. The amounts of ATP7A protein in lysates from G860V^C^ and G1255R^C^ fibroblasts grown at 30 °C or 37 °C, respectively, were therefore compared by WB (Fig. 6a). We included fibroblasts with ATP7A substitutions C1000R^C^ or A1362V^A^ (Fig. 6b), as growth at the reduced temperature has previously been shown to increase accumulation of C1000R and A1362D^34^. While decreased amounts of wild-type ATP7A were observed in fibroblasts from a healthy control and for the G1255R^C^ variant, an increased amount was observed for G860V^C^, (and for the positive controls C1000R^C^ and A1362V^A^) in fibroblasts grown at 30 °C rather than 37 °C (Fig. 6).

### Proteasomal degradation affects accumulation of G860V

**Figure 7:**
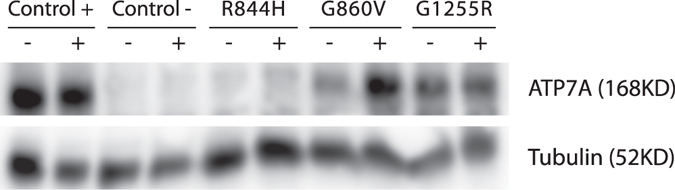
WB of lysates from MD fibroblasts cultured in the absence or presence of 25 µM Bortezomib, respectively. The MD fibroblasts contain different missense mutations as indicated. Control + is lysate from normal fibroblasts. Control − is lysate from MD fibroblast, containing a big deletion of the *ATP7A* gene (exon 3–23 deleted). The protein product of the housekeeping gene Alfa-Tubulin was used as an internal control for the correct loading of 25 μg protein in each lane.

To test whether proteasomal degradation is either fully or only partly responsible for the reduced accumulation of G860V^C^ and G1255R^C^, we treated the fibroblasts with the proteasome inhibitor Bortezomib for 20 hours, and determined the ATP7A protein content by WB. The data in Fig. 7 show that proteasomal degradation is involved in the degradation of G860V^C^, as proteasomal inhibition increased the accumulation of G860V^C^ protein, whereas no effect was observed for G1255R^C^. The variant R844H^C^ was also included and - as expected - no protein was observed, thereby supporting the notion that for this mutant, the absence of protein is due to unstable *ATP7A* transcript.

### Bypassing RNA splicing, revealed that mutations affecting RNA splicing do not affect the ATP7A activity per se

Missense mutations might cause disease because of aberrant *ATP7A* transcript splicing, reduced stability of the transcript and/or protein, or reduced Cu-ATPase activity as a result of the amino acid substitution *per se*. To distinguish between these effects, and to test for a possible correlation between cellular localization and activity of the ATP7A variants in fibroblasts, we used a yeast complementation assay based on a strain lacking the single *ATP7A* orthologue, *CCC2*, to determine *in vivo* activity of all 36 missense mutations. The assay is based on the fact that a *ccc2Δ* yeast host is unable to thrive under iron-limited conditions, while growth can be rescued by the expression of wild-type *ATP7A*.

**Figure 8:**
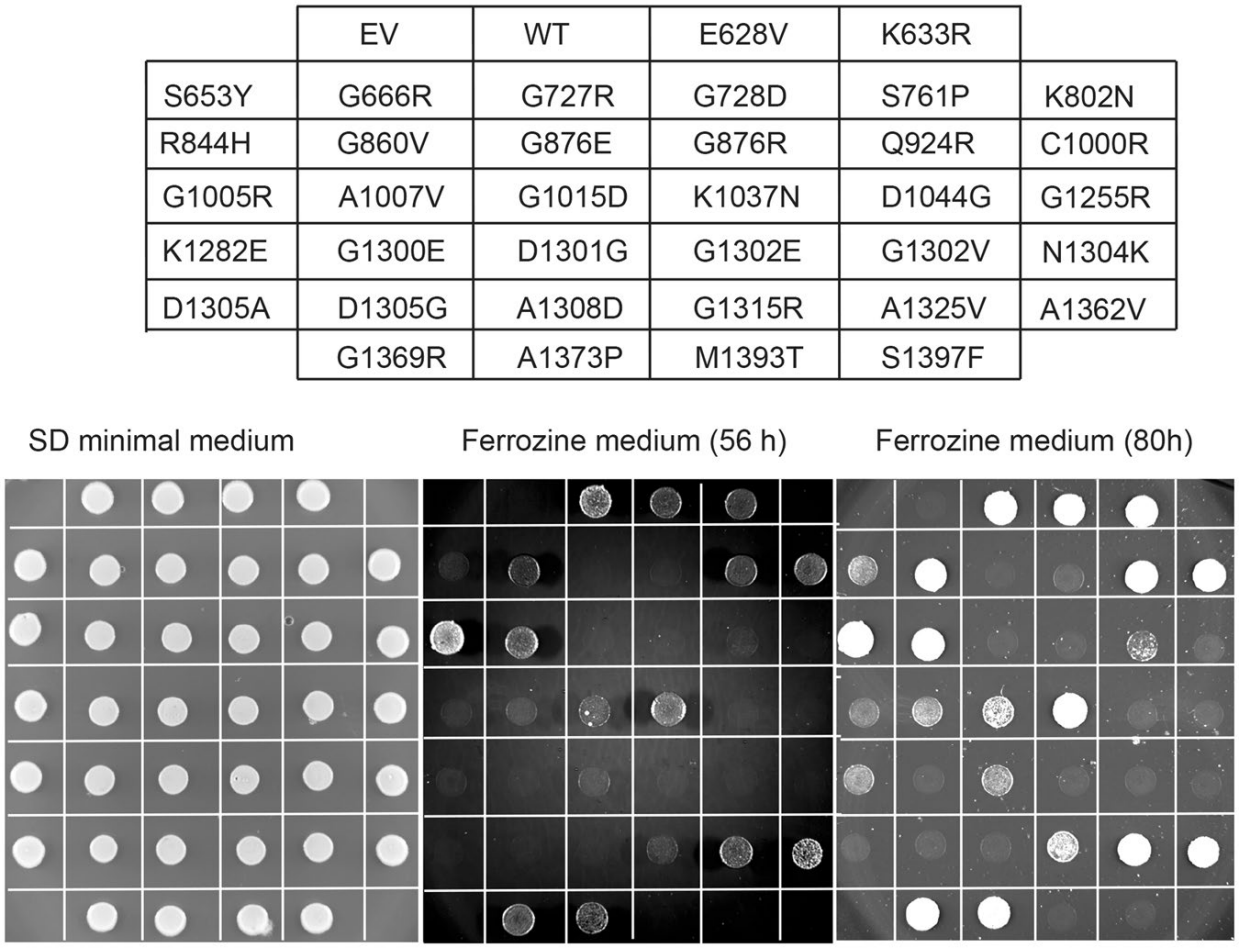
Complementation of the *ccc2Δ* iron requiring phenotype on agar plates. All 36 variants were investigated for their ability to complement the high iron requirement of a *ccc2Δ* yeast strain by plating cells on agar plates containing the iron chelator Ferrozine. WT, *ccc2Δ* yeast cells producing wild-type ATP7A; EV, *ccc2Δ* yeast cells expressing no ATP7A (empty vector). Mutants are spotted according to their amino acid substitution. To control for cell viability, each yeast strain was also spotted on iron-containing agar plates.

Seven of the studied mutations caused mal-splicing of *ATP7A* RNA in patient fibroblasts (Fig. 5). By introducing each mutation into the full length *ATP7A* cDNA sequence, the effect on RNA splicing was bypassed, allowing the investigation of the amino acid substitution *per se*. Six of the seven mutants, E628V^C^, K633R^C^, K802N^C^, K1037N^C^, G1369R^C^ and A1373P^C^ were able to complement the high iron requirement of the *ccc2Δ* yeast strain, comparable to the wild-type, indicating that these amino acid substitutions did not compromise the Cu-ATPase activity in itself (Fig. 8). Of the seven mutants, only G876R^C^ failed to show any *in vivo* activity.

Also R844H^C^ and G860V^C^ which resulted in a very small amount of *ATP7A* transcript and ATP7A protein in human fibroblasts, respectively, were able to complement the high iron requirement of the *ccc2Δ* yeast strain.

### Complementation of the ccc2 Δ yeast phenotype revealed poorer complementation of the TGN- trapped variants as compared to post-TGN mutants

Of the two mutants with copper dependent trafficking, the S761P^A^ substitution was able to complement the *ccc2Δ* yeast phenotype after 56 hours, whereas only faint complementation was observed for the Q924R^O^ substitution even after 80 hours. Investigation of the four mutations leading to post-TGN localization of the protein showed that substitutions G666R^A^, A1325V^A/O/C^ and G1015D^A^ were able to complement, whereas G876E^A^ was not. None of the 21 TGN-trapped variants were able to complement the *ccc2Δ* yeast strain as well as wild-type ATP7A. However, after 80 hours, complementation was observed for G1315R^C^ and A1362V^A^ and weak complementation for S653Y^A^, G1005R^C^, A1007V^A^, K1282E^A^ and D1301G^C^. No complementation was observed for the last 14 TGN-trapped variants: G727R^C^, G728D^A^, C1000R^A^, D1044G^C^, G1255R^C^, G1300E^C^, G1302V^C^, G1302E^C^, N1304K^C^, D1305A^C^, D1305G^C^, A1308D^C^, M1393T^C^ and S1397F^C^.

**Figure 9:**
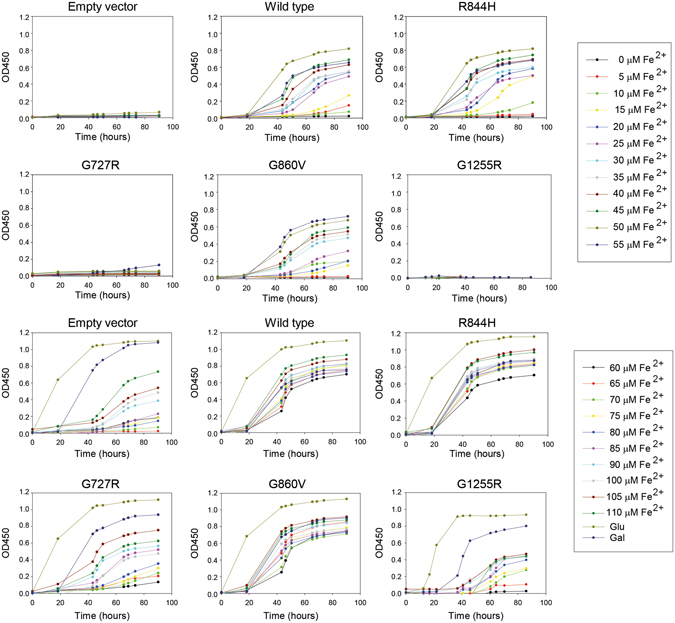
Complementation of the *ccc2Δ* iron requiring phenotype in liquid culture. The mutants R844H, G860V and G1255R leading to no, or only little protein accumulation in patient fibroblasts and the frequent mutant G727R were investigated for their ability to complement the high iron requirement of a *ccc2Δ* yeast strain in liquid cultures containing 1 mM Ferrozine and increasing amounts of Fe^2+^, as indicated. Wild-type, *ccc2Δ* yeast cells producing wild-type ATP7A; Empty vector, *ccc2Δ* yeast cells expressing no ATP7A.

Despite normal splicing, the mutation predicting the R844H^C^ substitution showed little accumulation of *ATP7A* transcript in human fibroblasts, while G860V^C^ and G1255R^C^ led to a reduced protein accumulation. To examine the activity of these variants in more detail, we analyzed their ability to complement the high iron requirement of the *ccc2* yeast knock-out strain at twenty different iron concentrations for 92 hours. The data in Fig. 9 show that R844H^C^ and G860V^C^ were able to complement to the same extent as the wild-type, whereas G1255R^C^ failed to show any complementation.

**Figure 10:**
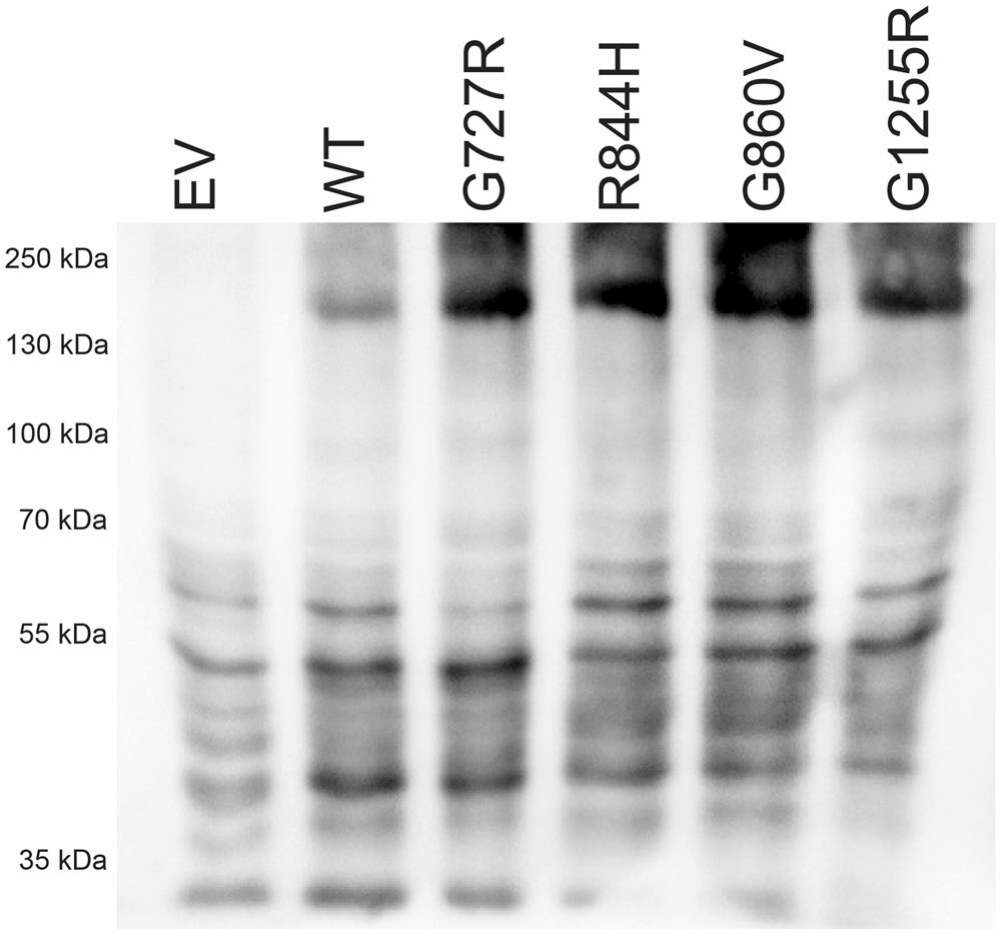
WB of crude lysates from yeast transformed with ATP7A expression plasmids. Crude membrane proteins were isolated from *ccc2Δ* yeast cells, transformed with plasmids encoding G727R, R844H, G860V, G1255R, WT, or no ATP7A (empty vector, EV). 25 μg crude membrane proteins were analyzed by Western blotting as described in Materials and Methods.

In contrast to the effect observed in fibroblasts, all three variants led to a substantial accumulation of protein in the yeast (Fig. 10), indicating that the disease-causing effect of the mutations predicting the R844H^C^ and G860V^C^ substitutions in humans is due to an unstable transcript and protein respectively, whereas the disease-causing effect of G1255R^C^ is primarily due to the lack of copper-transport activity. The frequent variant G727R^C^ found in about 4.7% of MD patients^3^ was also included. The observed absence of transport activity combined with a substantial amount of accumulated protein, verifies that the disease-causing effect of G727R^C^ indeed is due to the amino substitution *per se*. This does not concur with results published by Tang *et al*.^37^ who found a significantly reduced amount of G727R protein in patient fibroblasts, but also found that the G727R mutant was able to complement the *ccc2* yeast knock-out strain. The reason for this discrepancy is unknown. All lanes, including the lane representing the empty vector (EV, negative control) show some unspecific bands, whereas the band representing ATP7A is absent in the negative control.

## Discussion

**Figure 11:**
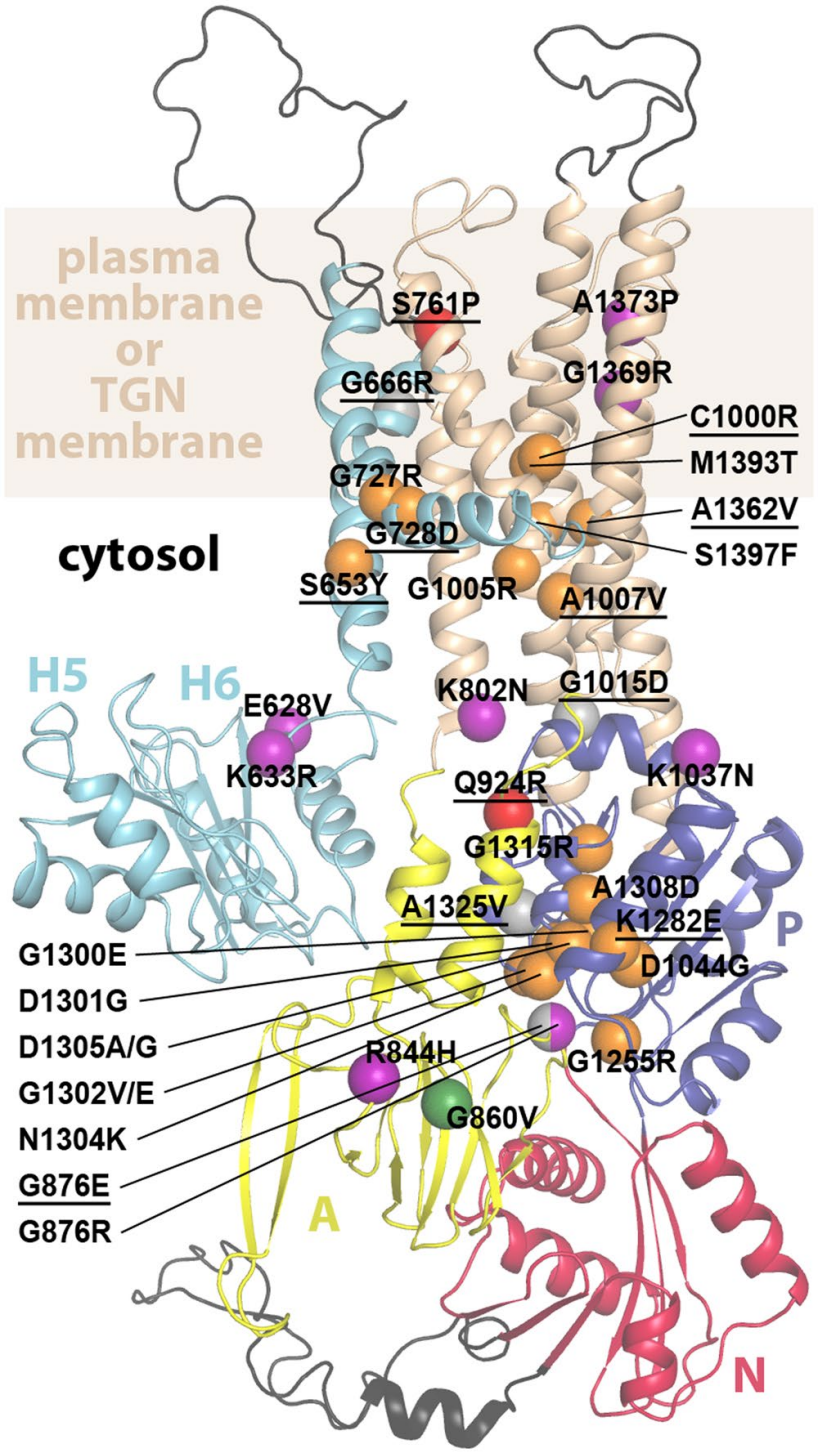
Distribution and effect of ATP7A disease-causing missense mutations. The mutations, presented in this study, are plotted as spheres in a previously established homology model of ATP7A^7^, based on the crystal structure of the homologous protein LpCopA from *Legionella pneumophila* ^3^. The various domains of ATP7A are colored as in Fig. 1. However, metal-binding domains 1 to 4 (H1–H4) are not depicted in the figure, as their structural localization is unknown, and regions with major insertions relative to LpCopA are shown in black. The approximate position of the (TGN- or plasma-) membrane is shown in wheat. The color of spheres represent the mutational effect on ATP7A localization: Copper dependent trafficking of the protein (blue); retention in post-Golgi compartments (grey); retention in the trans-Golgi network (orange); not certain (green); no detectable protein (purple). Underlined missense mutations have been reported to confer a non-classical phenotype. A1325V leads to classical MD in some patients (see Fig. 2).

We analyzed the effect of 36 *ATP7A* missense mutations on mRNA splicing, mRNA accumulation, protein accumulation, Cu-ATPase activity, cellular localization and copper-dependent trafficking. The observed effects of the 36 *ATP7A* missense mutations are summarized in Figs 2 and 11.

For eight of the 36 predicted amino acid substitutions - E628V^C^, K633R^C^, K802N^C^, R844H^C^, G876R^C^, K1037N^C^, G1369R^C^ and A1373P^C^ - no ATP7A protein was detected, and RT-PCR analyses revealed that all eight variants, except R844H^C^, affected splicing of the *ATP7A* transcript. The reason for the decreased amount of R844H^C^ transcript is puzzling. It is possible that this mutation enhances mRNA degradation by affecting the mRNA structure. The mal-splicing of the transcripts encoding E628V^C^ or K633R^C^ leads to the skipping of exon 8, while the mal-splicing of the transcripts that encodes G876R^C^ leads to the skipping of exon 12. These events result in a frameshift and a premature termination codon (PTC) after 34, or seven aberrant amino acids, respectively. The reduced amount of transcript observed in these cells may therefore be explained by the activation of nonsense-mediated decay (NMD)^38^. NMD is a translation-coupled quality control system that recognizes and degrades mRNA containing a PTC. The NMD complex is anchored to the exon junction complex (EJC), which is removed from the mRNA by the elongating ribosomes.

Skipping of exon 21 in G1369R^C^ and A1373P^C^ also leads to an out-of-frame transcript, but the PTC is located 25 nt upstream from the last exon, exon 23. The normal amount of transcript observed for these two mutants therefore concur with the notion that only PTC’s located more than 50–55 nt upstream from the last exon mediate NMD^38^. NMD is most likely not activated for K802N^C^ and K1037F^C^ as the skipping of exons 10 and 15, respectively, preserves the reading frame (Table 1).

As substitutions E628V^C^, K633R^C^, K802N^C^, R844H^C^, K1037N^C^, G1369R^C^ and A1373P^C^ were able to complement the *ccc2Δ* yeast strain, the absence or insufficient amount of correctly spliced full-length transcript is therefore the most likely cause of the classical MD phenotype observed in these 7 patients. Of the eight mutants, only G876R^C^ was unable to complement *ccc2Δ*, indicating that this substitution also affects the catalytic activity of ATP7A if some residual protein is properly produced; G876 is located in the TGE-loop of the A-domain which is responsible for dephosphorylation^39, 40^.

The two mutations predicting the substitution G860V^C^ and G1255R^C^, lead to a decreased accumulation of ATP7A protein in fibroblasts, despite the presence of normal amounts of correctly spliced mRNA, thus pointing to compromised protein folding. Indeed, the more bulky valine and arginine side chains may prevent formation of the central β-sheet of the A-domain, and decrease flexibility in a turn of the P-domain adjacent to the phosphorylation site, respectively. The reduced amount of accumulated G860V^C^ could be rescued at 30 °C and by inhibiting proteasomal degradation, indicating that the reduced amount of G860V^C^ seems to be controlled through proteasomal degradation. This was not the case for G1255R^C^, as neither reduced temperature nor inhibition of proteasomal degradation rescued the reduced accumulation of this variant.

The intracellular localization of ATP7A was affected in the majority of the investigated fibroblasts. Copper-dependent trafficking was only obtained for Q924R^O^ and S761P^A^. Q924 is adjacent to the amino-terminus of TMA where the MBDs are likely to interact through charge-complementation. The glutamine-to-arginine substitution may thus compromise the interaction of the MBD-complex with the catalytic core of the protein. S761 is structurally located near D782, which corresponds to E189 that is implicated in copper release in *L*. *pneumophila* CopA^5^. Considering that the two mutations result in a non-classical phenotype and OHS, respectively, and that they display some complementation, these protein variants are quite likely to be partly functional in the patients.

21 of the mutants (S653Y^A^, G727R^C^, G728D^A^, C1000R^A^, G1005R^C^, A1007V^A^, D1044G^C^, G1255R^C^, K1282E^A^, G1300E^C^, D1301G^C^, G1302V^C^, G1302E^C^, N1304K^C^, D1305A^C^, D1305G^C^, A1308D^C^, G1315R^C^, A1362V^A^, M1393T^C^, and S1397F^C^) prevented copper-induced trafficking from the TGN to cytoplasmic vesicles or the plasma membrane, whereas only four of the mutants (G666R^A^, G876E^A^, G1015D^A^, A1325V^A/O/C^), led to permanent post-TGN, cytoplasmic localization.

The D1044E substitution has previously been shown to inhibit trafficking from TGN^12, 13^ while a mutated ^875^TGE motif (replaced by AAA) leads to permanent localization of the ATP7A protein to the plasma membrane, regardless of the copper concentration^13^. These observations together with the fact that D1044 is essential for phosphorylation and ^875^TGE is required for de-phosphorylation, led to the hypothesis that copper-regulated trafficking of ATP7A from TGN to the cell membrane is associated with the formation of the phosphorylated catalytic intermediate, whereas retro-trafficking from the cell membrane to TGN is associated with dephosphorylation^13^. Furthermore it has been shown that mutation of the conserved aspartic acid residue in ATP7B blocks redistribution from TGN, whereas mutation of the conserved TGE phosphatase domain traps ATP7B in cytosolic vesicular compartments. Nevertheless, this putative coupling between phosphorylation/dephosphorylation and cellular localization is still being debated^41, 42^.

We observed that mutants with retained TGN localization, which are typically linked to the classical MD phenotype, and with no or poor *ccc2Δ* yeast complementation (note that among these, mutants with some capacity to complement represent exceptions with less severe phenotypes), generally cluster within domains that are crucial for phosphorylation (Figs 2 and 11). One group may impair phosphorylation indirectly as the delivery of copper to the essential TM copper-binding site(s) is required for the completion of phosphorylation, whereas other residues compromise phosphorylation more directly. Specifically, substitutions S653Y^A^, G727R^C^, G728D^A^, C1000R^A^, G1005R^C^, A1007V^A^, A1362V^A^, M1393T^C^ and S1397F^C^ target the TM-domain and may affect copper-uptake at the GG- platform (S653Y^A^, G727R^C^ and G728D^A^, located in TMA and TMB) or copper-transport directly by affecting ion-binding to the intramembranous site(s) (C1000R^A^, M1393T^C^, S1397F^C^ and A1362V^A^)^5^. The observed cellular location of A1362V^A^ concurs with a previous publication^27^. The mutants D1044G^C^, G1255R^C^, G1300E^C^, D1301G^C^, G1302V^C^, G1302E^C^, N1304K^C^, D1305A^C^, D1305G^C^, A1308D^C^ and G1315R^C^ all target the P-domain and may thus prevent formation of the acyl-phosphate intermediate with D1044.

**Figure 12:**
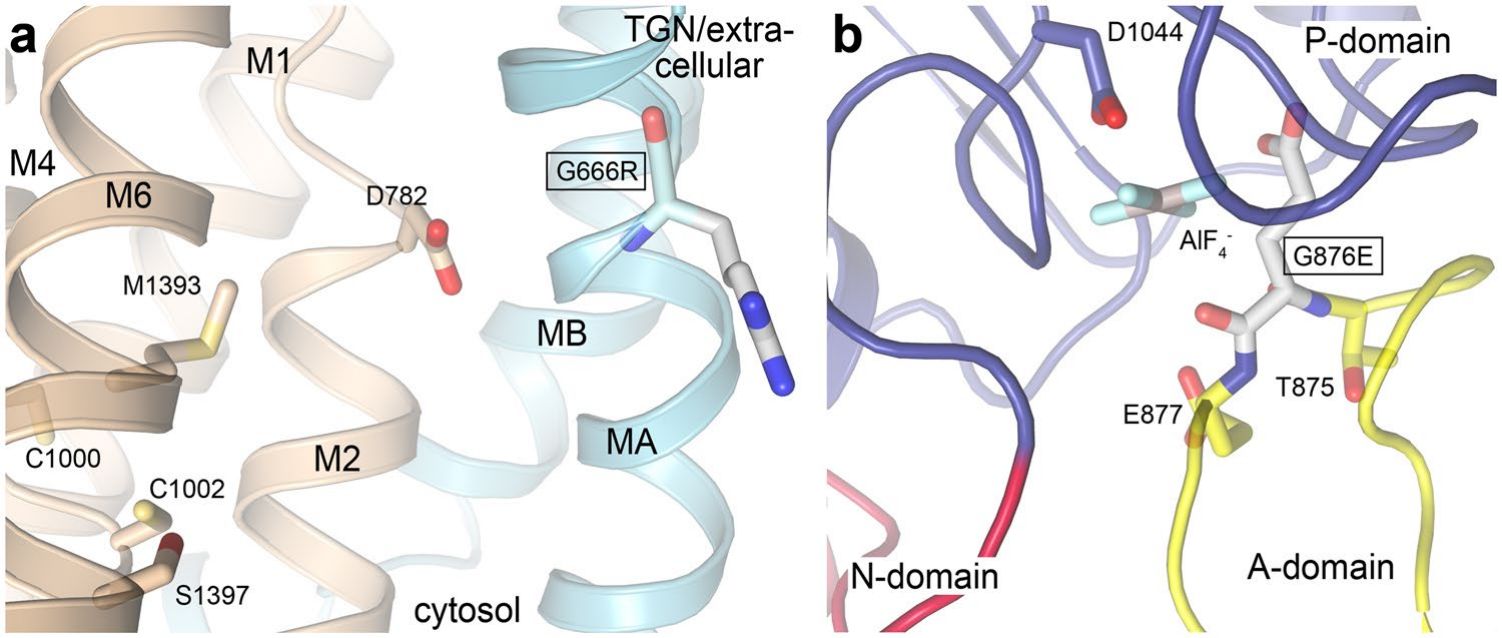
The post-Golgi arrested mutations G666R^A^ and G876E^A^. The domains of ATP7A are colored as in Fig. 1, and functionally relevant residues shown as sticks using a previously established homology model of ATP7A (6) based on the crystal structure of the homologous protein LpCopA from *Legionella pneumophila* (2). **(A)** close-view of G666R at the possible release pathway from the CPC-motif (C1000 and C1002) and via D782 to the non-cytosolic side. The mutation may directly prevent copper passage through sterical hindrance or influence of the local environment at the membrane interface. (**b**) Close-view of G876E at the catalytic Aspartate (D1044). G876 is located in the TGE-loop of the A-domain which is responsible for dephosphorylation (AlF_4_^−^ is a phosphate mimic used for structure determination of LpCopA). The mutation is likely to prevent dephosphorylation and turn-over.

In contrast, none of the four substitutions G666R^A^, G876E^A^, G1015D^A^, A1325V^A/O/C^ were detected in TGN under any conditions, although WB confirmed the presence of normal levels of ATP7A protein in the cells. These mutants have less severe effects on patients and are able to complement in *ccc2Δ* yeast (except in the case of A1325V^A/O/C^). This may indicate hyper- phosphorylation of the proteins with permanent localization to the cytoplasmic vesicles or plasma membrane. Indeed, the structural localization of these residues supports this notion to some degree. G666 is located in TMA adjacent to the copper exit site formed partly by D782, which corresponds to E189 in *L*. *pneumophila* CopA^5^, and possibly hampers copper-release and de-phosphorylation (Fig. 12a). G876 and A1325 are located near the phosphorylation site (Figs 11 and 12b) and may influence de-phosphorylation negatively; in fact, G876 is, as described above, the second amino acid of the conserved TGE-motif in the A-domain, responsible for de-phosphorylation. Finally, G1015 is located in the cytoplasmic linker between M4 and the P-domain and might abolish de-phosphorylation by preventing the coupling of the TM- and P-domains which is essential for the catalytic cycle. However, we cannot exclude the possibility that G1015D^A^ and A1325^A/O/C^ might obstruct phosphorylation as they are positioned in or close to the P-domain.

It is probably not the phosphorylation status *per se* that determines the localization of ATP7A, but rather the concomitant conformation (according to the above mentioned E1-E1P-E2P-E2 reaction) with considerably altered three-dimensional structures throughout the reaction cycle (re-visit Fig. 1). The reason why E2 states (associated with de-phosphorylation) may be dominant when ATP7A is in TGN, and E1 conformations (associated with phosphorylation) appear linked to post-TGN localization, could be that distinct conformations have different affinities to trafficking-assisting molecules; this would be highly interesting considering that the catalytic turnover is relatively fast^41^, whereas trafficking takes much longer, but is in agreement with the novel concept of resting functional states of wild-type P-type ATPase transporters^43^.

It is noteworthy that all mutations leading to copper dependent trafficking or plasma membrane localization and/or trapping of the protein in cytoplasmic vesicles have been identified in patients with an atypical phenotype or OHS (6/6), indicating residual protein activity. The presence of residual activity of these mutants was supported by complementation of the *ccc2Δ* yeast strain, which showed that five out of six were able to complement the *ccc2Δ* yeast strain. In contrast, most of the mutations leading to permanent TGN localization of the protein conferred classical MD (71%; 15/21). Furthermore, only seven out of 21 (33%) TGN-trapped variants were able to complement the *ccc2Δ* yeast strain, and four of these conferred a mild phenotype. All seven had reduced activity; growth was only visible after many hours of incubation. Altogether, the *ccc2Δ* yeast complementation results support the notion that development of a mild phenotype is associated with a partially active protein.

In MD, copper accumulates in intestinal cells leading to copper deficiency in the body and subsequently to reduced cuproenzyme activity. Some cuproenzymes are located in the mitochondria (cytocrome c oxidase), others in the cytosol (tyrosinase and Cu/Zn superoxide dismutase) and some are secretory enzymes loaded with copper in TGN (lysyl oxidase, ceruloplasmin, dopamine ß-hydroxylase)^44^. One explanation for the milder phenotype of patients with an ATP7A variant located at the plasma-membrane or trapped in cytoplasmic vesicles, as opposed to a TGN-located variant, could be that the plasma membrane/vesicles located variants are able to transport some copper from the intestine to the blood stream, thereby enabling some copper-loading of cuproenzymes located in the mitochondria or the cytosol. The reason why e.g. OHS patients with small amounts of normal protein that stem from splice site mutations, have pronounced connective tissue defects, due to the absence of active lysyl oxidase, is puzzling. As proposed by Mercer^45^, it could also be explained by the ability of the small amount of ATP7A to export copper to the blood stream, whereas transport of copper to TGN might be hampered. MD fibroblasts accumulate copper, and the small amount of wild-type protein might be occupied at the plasma membrane to excrete copper, due to high cellular copper.

Whereas the cellular localization might predict the severity of the disease, it is unlikely that it correlates with the effect of copper treatment. Early treatment rather than the exact character of the mutation seems to be the most important factor for the outcome. A significant effect of early copper treatment of patients even with predicted severe mutations has been demonstrated in several publications^46–48^. The treatment of three patients with the frequent G727R variant led to a near normal outcome in two of the three patients who were treated within 25 days after birth, in contrast to the poor outcome in the third patient who began treatment at 7.6 months^48^. Similarly, 35 MD patients with different mutations, who were treated before the age of 1 month led to a significant improvement and a higher survival rate in 25 of the 35 patients (71.2%) after the age of 3 years. Poorer improvement and a lower survival rate of only 50% after the age of 3 years^48^ was observed in a group of 22 patients whose treatment was initiated later than 1 month after birth. The 35 patients who were treated early had a mixture of some expected mild and some severe mutations (e.g Q724H, del Ex1, del Ex2–14, del Ex20-23, G666R, G728D, 2233delT, Q724X and A629P). Similar cases of mixed mutations were observed in the 22 patients who were treated later (e.g Q724H, del Ex1, del Ex13-14, K1034N and Q1383X)^48^.

In summary, most substitutions that lead to TGN-trapping have no or low catalytic activity, and predispose to a severe classical MD phenotype, whereas most variants that lead to copper dependent trafficking or to cytoplasmic localization, have residual catalytic activity and render a milder phenotype. The mechanism behind the trafficking is very complex. We found that several ATP7A substitutions assumed to affect the catalytic phosphorylation and located in two different domains, led to permanent TGN localization, whereas substitutions that may influence dephosphorylation impair TGN-retrieval. These results support previous studies indicating that phosphorylation is crucial for the exit of ATP7A from TGN, while dephosphorylation is crucial for recycling from the cytoplasmic compartments back to TGN^13^. In contrast to the previously published paper that studied cells transfected with plasmids encoding ATP7A variants^13^ we used a setup based on a large number of endogenously expressed ATP7A variants in fibroblasts from MD patients. The present results verify and add to previous suggestions.

Furthermore, in contrast to other studies, we also investigated the effect of missense mutations on the ATP7A transcript and demonstrate that a substantial number of predicted missense mutations found in MD patients prohibit correct exon-intron splicing, while the amino acid substitution *per se* does not abolish Cu-ATPase activity. These are new observations which contribute significantly to our understanding of MD.

## Methods

### Patients

The 36 patients were referred to the Kennedy Center for diagnostic confirmation of Menkes disease. We divided the patients into three sub-classes based on phenotype and progression: Classical Menkes disease (C) for patients with severe symptoms and death before 5 years of age; Atypical MD for patients with milder symptoms and longer survival (more than 5 years). The Occipital Horn Syndrome (OHS) for patients with mainly connective tissue manifestations. We have previously^29^ described the patient with the K1283E mutation as classic. However, we now know that the patient survived for at least 15 years and have therefore re-assigned this case to Atypical MD.

### Fibroblast culturing

Fibroblast cell cultures obtained from patient-skin biopsies (and one control) were cultured as described previously^49, 50^.

### Proteasomal inhibition

Fibroblast cell cultures were grown in the presence of 20 µM Bortezombib (added to the normal medium) for 20 hours before harvesting.

### Copper-uptake (^64^Cu) and –retention

The copper-uptake (^64^Cu) and retention profile of the fibroblast cultures were performed as described previously^50^.

### Immunofluorescence

Immunofluorescence (IF) was performed as described previously^29^. In brief, fibroblasts grown on glass coverslips were incubated in pure F-10 with 50 µM Bathocuproine disulfonic acid (BCS; Sigma; www.sigmaaldrich.com) for 2 hours. The cells were subsequently fixed in freshly prepared 4% paraformaldehyde in PBS for 20 min at 4 °C, washed and permeabilized with 0.2% Triton-X100 in PBS. Nonspecific signals were blocked with 3% BSA in PBS with 0.2% Triton and subsequently incubated with chicken anti-ATP7A antibodies against amino acids 1407–1500 (ab 13995, Abcam, www.abcam.com). In parallel, the cells were incubated with mouse antibodies against the 28-kDa SNARE protein (GS28) located in the Golgi compartment (BD transduction laboratory, www.bdbiosciences.com). Alexa 488 conjugated goat anti-chicken secondary and tertiary Alexa 488 rabbit anti-goat antibodies were used to detect the ATP7A protein, whereas Alexa 546 conjugated donkey anti-mouse secondary antibodies were used to detect GS28. The localization of ATP7A was analyzed with confocal microscopy (Olympus 1000 Fluoview) and the degree of co-localization was analyzed by Coloc 2 (Fiji/http://imagej.net/ImageJ).

### Western blotting

Cultured patient fibroblasts were harvested by trypsination and lysed in lysis-buffer (50 mM Hepes pH 7.6; 250 mM NaCl; 0.1% NP40; 5 mM EDTA) for 30 minutes on ice. The protein concentration in the lysates was determined using the BCA protein assay kit (Pierce, Rockford, IL, USA). A sample corresponding to 25 µg protein was mixed with loading-buffer and DTT, and loaded on 12-well RunBlue SDS gels (Expedeon, www.expedeon.com/). SDS-PAGE was carried out at 130 V and the Western blot was performed according to the manufacturer's protocol (Expedeon, www.expedeon.com/). After blotting, the membranes were blocked for one hour in blocking-buffer (ATP7A: 25 mM Tris-buffer (pH 7.4); 5% skimmed milk; 0.5% Tween20. GAPDH: 25 mM Tris-buffer (pH 7.4); 5% skimmed milk; 0.1% Tween20; 0.25 M NaCl). The membranes were cut in two so that the upper part contained the ATP7A protein (168 KD) and the lower part contained the GAPDH protein (38 KD). Thereafter, the membranes were incubated with primary antibody diluted in incubation buffer (25 mM Tris-buffer (pH 7.4); 5% skimmed milk; 0.1% Tween20) overnight at 4 °C. The primary antibodies were chicken polyclonal anti-ATP7A (ab13995, abcam; 1:1,000) and rabbit polyclonal anti-GAPDH (NB300-327, Novus biological; 1:5000), respectively. The next day the membranes were washed for 8 × 5 minutes in washing buffer (10 mM Tris-buffered saline (50 mM Tris, 150 mM NaCl, pH 7.6), 0.1% Tween20). Secondary antibody diluted 1:5,000 in washing buffer was then added to the membranes which were left to incubate for one hour at room temperature on a shaking stand. The secondary antibodies used were donkey anti-chicken (HRP) (DAKO, www.dako.com/dk/) and swine anti-rabbit (HRP) (DAKO, www.dako.com/dk/) to target the primary antibodies against ATP7A and GADPH, respectively. The membranes were washed again for 8 × 5 minutes in washing buffer. The HRP signal was detected using the Super Signal West Dura kit according to the manufacturer’s protocol (Pierce; Rockford, IL, USA) and visualized by autoradiography using Amersham Hyperfilm ECL (Amersham, www.gelifesciences.com).

For Western blotting of yeast-produced ATP7A, cells were inoculated at 30 °C to OD_450_ = 0.05 in minimal medium with galactose as the sole carbon source. Cells were harvested at OD_450_ = 1.0 and crude membranes were prepared as described previously^51^. SDS-PAGE and Western blotting using the same polyclonal anti-ATP7A antibody and HRP conjugated donkey anti-chicken antibody used above were performed as described^52^ and visualized using the chemiluminescent Immobilon kit from Millipore and the LAS4500 imager from GE Healthcare.

### Quantitative Real-time PCR

Real-time PCR was performed on cDNA. A Taq-Man 6-carboxy-fluorescein (FAM) labeled probe and primer pairs against the boundary between exon 1 and exon 2 (part number Hs00921963_m1) or against the boundary between exon 22 and exon 23 (part number Hs00921966_m1) in *ATP7A* cDNA were used to detect the total amount of *ATP7A* transcript. A FAM labeled probe and primers for the human *GAPDH* transcript (part number 4352934E) were used as an endogenous control. Relative quantification of *GAPDH* transcript was carried out on parallel samples. The cDNA samples obtained from the RNA preparations were assayed in triplicate, in 100 ng/sample, in a total volume of 25 μl. All probes were purchased from Applied Biosystems. PCR amplification and detection were performed with an ABI7500 (Applied Biosystems, www.invitrogen.com) in accordance with the manufacturer’s instructions. The threshold cycle (CT) is defined as the fractional cycle number at which the fluorescence passes a fixed threshold. Standard curves for CT values compared with log cDNA concentration were prepared by assaying five-fold serial dilutions of control cDNA: from 100 ng/sample to 0.16 ng/sample, with the *GAPDH* and *ATP7A* probes, respectively. In all experiments, the amount of *ATP7A* and *GAPDH* mRNA were calculated by linear regression of the lines generated by the standard curves, log cDNA concentration against CT. The amount was measured as the relative amount compared to a control sample. The normalized *ATP7A*_*N*_ value is calculated by dividing the *ATP7A* mRNA value with the *GAPDH* value (SDs are shown).

### Investigation of ATP7A transcript by RT-PCR

Total RNA from approximately 5 × 10^6^ cultured skin fibroblasts was isolated with the RNAeasy kit (Qiagen, www.qiagen.com), and single-stranded cDNA was synthesized with the High-Capacity cDNA Archive Kit in accordance with the manufacturer's instructions (Applied Biosystems, www.invitrogen.com). PCR amplification of fragments spanning the cDNA fragments of interest - including the affected and flanking exons - was performed with sequence-specific primers as noted in the legends to Fig. 5. PCR products were subsequently separated and visualized upon agarose-gel electrophoresis in the presence of Ethidium Bromide, excised from the gel and purified with the QIAquick gel extraction kit (Qiagen, www.qiagen.com) and sequenced using the PCR amplification primers. Sequencing was performed using an ABI3100 sequencer (Applied Biosystems, www.invitrogen.com). The sequence of used primers is shown in Supplementary Table 2.

### Yeast expression plasmids and complementation test

Site-directed mutagenesis of human Menkes cDNA was performed according to ref. 53. Menkes expression plasmids were constructed by homologous recombination by transformation of *Hin*dIII, *Sal*I, *Bam*HI digested pEMBLyex4^54^ and wild-type or mutated human Menkes cDNA into strain PAP6064 (*mat a his3 Δ1::UPR-lacZ HIS3 leu20 met15 Δ0 ura3v Δ0 ccc2::kanMx4*) as described previously^55^. The nucleotide sequences of all constructs were confirmed by DNA sequencing. Complementation tests were performed by spotting 5 µl of exponentially growing cells at OD_450_ = 0.5 onto SG (minimal medium with galactose) plates with 1 mM Ferrozine, 1 µM CuCl_2_ and 135 µM Fe(NH_4_)_2_SO_4_. To confirm viability, 5 µl of each yeast culture was also spotted on non-selective medium (SD minimal medium). The plates were incubated at 30 °C and inspected daily. For determining complementation in liquid culture, cells were grown on micro plates in media containing 1 mM Ferrozine, 1 µM CuCl_2_ and Fe^2+^ concentrations ranging from 0–105 µM (in the form of Fe(NH_4_)_2_SO_4_) as indicated in Fig. 9.

### Study approval

All experiments were carried out in accordance with relevant guidelines and regulations. The cell lines and patient information were previously published^5, 29^.

## Electronic supplementary material

Supplementary files
