# Supplementary material for: Characterization of ATP7A missense mutants suggests a correlation between intracellular trafficking and severity of Menkes disease
Source: Sci Rep. 2017 Apr 7;7:757. doi: 10.1038/s41598-017-00618-6 (PMC5428812; doi:10.1038/s41598-017-00618-6)
Supplement: Supplementary file 1 — Supplementary files [file 41598_2017_618_MOESM1_ESM.pdf]

Supplementary files

(Table 1, 2; Figure 1-4)

Characterization of ATP7A missense mutants suggests a correlation between intracellular trafficking and severity of Menkes disease.

Tina Skjørringe[1,2¥], Per Amstrup Pedersen[3¥], Sidsel Salling Thorborg[1], Poul Nissen[4], Pontus Gourdon[5,6], Lisbeth Birk Møller[1,7*].

Fibroblast culture, treatment	Pearson´R value (above threshold)
Neg BCS	-0.02
Neg Cu	-0.19
Pos BCS	0.51
Pos Cu	0.07
Q924R BCS	0.41
Q924R Cu	0.06
S761P BCS	0.18
S761P Pro Cu	-0.06
S653Y BCS	0.39
S653Y Cu	0.47
G727R BCS	0.30
G727R Cu	0.42
G728D BCS	0.48
G728D Cu	0.44
C1000R BCS	0.52
C1000R Cu	0.46
G1005R BCS	0.59
G1005R Cu	0.57
A1007V BCS	0.39
A1007V Cu	0.54
D1044G BCS	0.63
D1044G Cu	0.49
G1255R BCS*	0.12
G1255R Cu	0.50
K1282E BCS	0.48
K1282E Cu	0.29
G1300E BCS	0.59
G1300E Cu	0.60
D1301G BCS	0.30
G1301G Cu	0.50
G1302V BCS	0.34
G1302V Cu	0.37
G1302E BCS	0.73
G1302E Cu	0.71
D1304K BCS	0.59
D1304K Cu	0.75
D1305A BCS	0.59
D1305A Cu	0.57
D1305G BCS	0.50
D1305G Cu	0.59
A1308D BCS	0.57
A1308D Cu	0.63
G1315R BCS	0.32
G1315R Cu	0.53
A1362V BCS	0.59
A1362V Cu	0.56
M1393T BCS	0.47
M1393T Cu	0.63
S1397F BCS	0.54
S1397F Cu	0.53

Supplementary Table 1. The Pearson's correlation R values obtained by investigation of the IF pictures shown in Fig. 3, using the colocalization analysis tool, Coloc 2 from Fiji. An R value of 1 is total positive correlation, 0 is no correlation, and −1 is total negative correlation.* This low value might be due to very low expression level. Noise affect the R value negatively.

Primers and sequences	
Exon 7 Forward (2138F)	5´-cctggcaaccaacaaagcaca-3´
Exon 14 Reverse (3192C)	5´-tccataccaagagggtggcaat-3´
Exon 12 Forward (2900F)	5´-aggaggcaaatttccagtgga-3´
Exon 17 Forward (3680F)	5´-aggttgtgccaggctgtggtatt-3´
Exon 19 Forward (4044F)	5´-cctgaagcagaactggctatccatat-3´
Exon 23 Reverse (4611C)	5´-actcttctgtcctatctggctccg-3´
Primers used for RT-PCR amplification	
Exon 7-Exon 14	2138F + 3192C
Exon 12-Exon 23	2900F + 4611C
Exon 19-Exon 23	4044F + 4611C
Exon 17-Exon 23	3680F + 4611C

Supplementary Table 2. Location and sequence of used primers used for RT-PCR investigation.

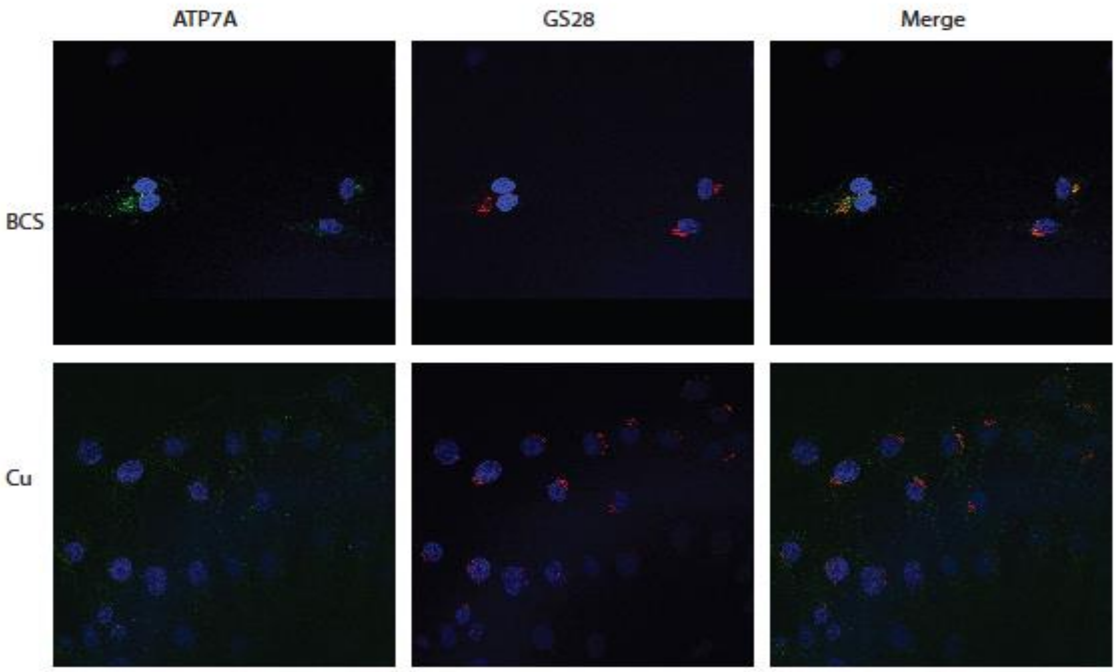

Supplementary Figure 1. Cellular investigation of endogenous ATP7A protein in control fibroblasts by indirect immunofluorescence (IF), additional pictures. Normal control cells (C+) were stained with primary antibodies against ATP7A (green; position 1) and the Golgi specific marker GS28 (red; position 2), respectively. Also a merge picture is shown (position 3). The nuclei were counterstained with DAPI. Copper-dependent trafficking was investigated in the presence of BCS (upper panel) and $CuCl_2$ (lower panel), respectively.

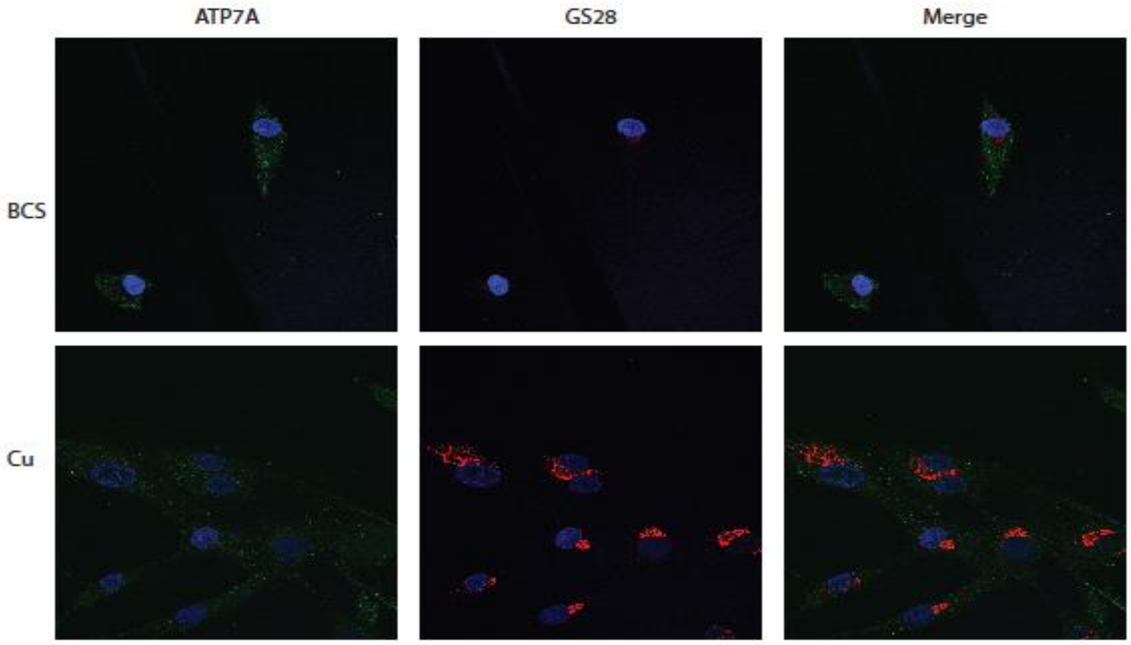

Supplementary Figure 2. Cellular investigation of negative control fibroblasts by indirect immunofluorescence (IF), additional pictures. Fibroblasts from a MD patient with a deletion of exons 3-23 (c.121-?_8333+?del), ATP7A negative control cells (C-) were stained with primary antibodies against ATP7A (green; position 1) and the Golgi specific marker GS28 (red; position 2), respectively. Also a merge picture is shown (position 3). The nuclei were counterstained with DAPI. Copper-dependent trafficking was investigated in the presence of BCS (upper panel) and $CuCl_2$ (lower panel), respectively.

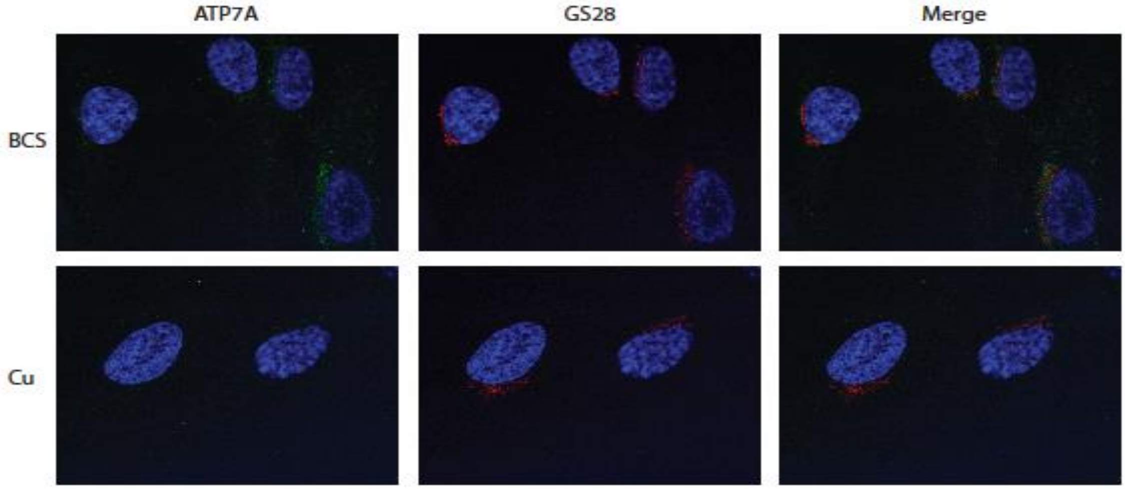

Supplementary Figure 3. Cellular investigation of endogenous S761P[A] ATP7A protein by indirect immunofluorescence (IF), additional pictures. Fibroblast expressing the endogenous mutant S761P[A] were stained with primary antibodies against ATP7A (green; position 1) and the Golgi specific marker GS28 (red; position 2), respectively. Also a merge picture is shown (position 3). The nuclei were counterstained with DAPI. Copper-dependent trafficking was investigated in the presence of BCS (upper panel) and $CuCl_2$ (lower panel), respectively.

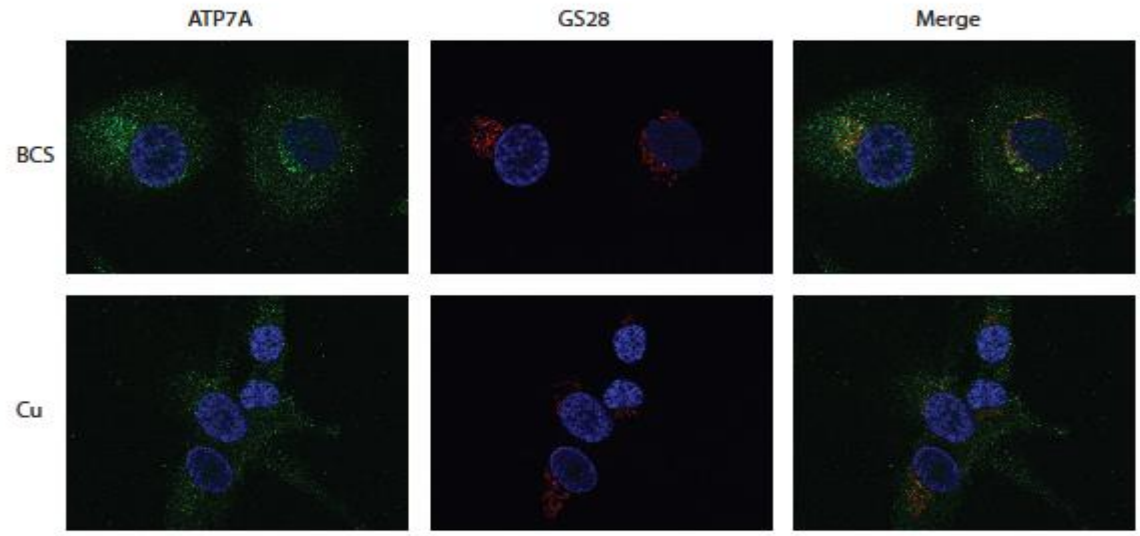

Supplementary Figure 4. Cellular investigation of endogenous Q924R^{O} ATP7A protein by indirect immunofluorescence (IF), additional pictures. Fibroblast expressing the endogenous mutant Q924R^{O} were stained with primary antibodies against ATP7A (green; position 1) and the Golgi specific marker GS28 (red; position 2), respectively. Also a merge picture is shown (position 3). The nuclei were counterstained with DAPI. Copper-dependent trafficking was investigated in the presence of BCS (upper panel) and $CuCl_2$ (lower panel), respectively.
